# The effect of an integrated reading and anxiety intervention for poor readers with anxiety

**DOI:** 10.7717/peerj.10987

**Published:** 2021-02-24

**Authors:** Deanna Francis, Jennifer L. Hudson, Saskia Kohnen, Lynn Mobach, Genevieve M. McArthur

**Affiliations:** 1 Macquarie University Centre for Reading, Department of Cognitive Science, Macquarie University, NSW, Australia; 2 Centre for Emotional Health, Department of Psychology, Macquarie University, NSW, Australia; 3 Behavioural Science Institute, Radboud University Nijmegen, Nijmegen, Netherlands

**Keywords:** Poor reading, Anxiety, Intervention case series, Treatment

## Abstract

A recent systematic review has reported that poor reading is reliably associated with anxiety. However, we currently lack evidence-based intervention for children who have both poor reading and anxiety (PRAX). In this study, we tested a new PRAX intervention in 8- to 12-year-old children using a double-baseline intervention case series design. Analyses of both group and individual data revealed that 12 weeks of PRAX intervention significantly improved children’s reading and spelling accuracy, and significantly reduced both anxiety disorders and symptoms. These results support PRAX intervention as a treatment for comorbid reading and anxiety problems in children and pave the way to a randomised controlled trial.

## Introduction

Around 16% of children find it difficult to learn to read compared to children who are the same age or in the same grade (Shaywitz et al., 1992). Some of these children have a persistent difficulty learning to read while other children fail to learn to read because of inadequate reading instruction. There are also some children with both a persistent reading difficulty and poor instruction. The reading problems that a child experiences may include a specific problem learning to read words accurately using the grapheme-phoneme correspondence (GPCs) rules (i.e. poor phonological recoding), while some struggle to learn to recognise whole words from memory (i.e. poor visual word recognition; Castles & Coltheart, 1996; Kohnen et al., 2018; Stuart & Stainthorp, 2016; Temple & Marshall, 1983). Others learn to read words accurately but struggle to read fluently (Lovett, 1984; Morris et al., 1998) or struggle to understand the meaning of what they read (poor comprehenders; Nation et al., 2010; Nation & Snowling, 1997; Oakhill, 1984). While many children have these specific reading problems, an even greater number have two or more of these difficulties (Castles & Coltheart, 1996; Goulandris & Snowling, 1991; McArthur et al., 2013; Peterson, Pennington & Olson, 2013). In addition, many of these children also struggle with their spelling (Landerl & Moll, 2010; Moll et al., 2014).

Another common childhood problem is anxiety, which affects around 10% of children (Copeland et al., 2014). Like poor reading, children have different types of anxiety. One of the most prevalent types of anxiety is social anxiety, which includes worries about performance situations such as reading aloud, meeting new children, or joining conversations (Lawrence et al., 2015). Other types of anxiety include generalised anxiety (i.e. numerous and persistent worries about school performance, friendships, family matters, or daily activities), separation anxiety (i.e. worry about separation from parents or home, distress before school, difficulty spending time alone), and specific phobias (i.e. fear of specific situations, objects, or animals such as dogs, as well as heights, doctors, or thunderstorms). Related anxiety problems include obsessive-compulsive disorder (i.e. repetitive or intrusive thoughts or behaviours) and panic disorder with or without agoraphobia (i.e. sudden and intense onset of fear with or without the fear of being unable to escape; Diagnostic and Statistical Manual 5th edition; DSM-5; American Psychiatric Association, 2013). Each type of anxiety falls along a continuum. Some children experience symptoms that meet diagnostic criteria for a disorder, while others experience symptoms that do not meet these criteria. There is high comorbidity between the anxiety disorders, with social, separation, and generalised anxiety often co-occurring in children (Kendall et al., 2010; Kendall, Brady & Verduin, 2001). Anxiety also shares a close comorbidity with depression particularly in adolescents and adulthood (Garber & Weersing, 2010).

Traditionally, poor reading and anxiety in children have been studied independently. However, in recent years, there has been growing concern that children with poor reading are at increased risk for anxiety (Carroll et al., 2005; Maughan & Carroll, 2006; Willcutt et al., 2013). This concern inspired a recent systematic review and meta-analysis of the association between poor reading and anxiety (Francis et al., 2019), which was found to be moderate and statistically reliable (*d* = 0.41), while the relationship between poor reading and depression was weak yet reliable (*d* = 0.23). This outcome raised the question of how comorbid problems in reading and anxiety might be reduced in children. While there are effective interventions for poor reading, and effective interventions for anxiety, there are no interventions that address both reading and anxiety at the same time. Indeed, no study has tested the efficacy of an intervention that combines treatment for both poor reading and anxiety. In fact, we can find only one peer-reviewed study that has tested an intervention for children with poor reading and anxiety, and this study provided intervention for just reading—not anxiety (Grills et al., 2014). Thus, the current study develops the first schema (e.g. an approach) to integrate reading and anxiety interventions for children with comorbid reading and anxiety difficulties. In the sections that follow, we review the existing evidence for available reading and available anxiety interventions for children.

The weight of existing evidence suggests that an intervention programme for reading or anxiety should cater for the heterogeneous needs of individuals by providing targeted treatment for each of those needs (Chorpita, Becker & Daleiden, 2007; Coyne, Kame’enui & Simmons, 2004). Thus, a PRAX intervention schema should include a suite of ‘modules’ that target this range of needs. For reading, the evidence suggests that these modules aim to improve phonological decoding, visual word recognition, reading fluency, and reading comprehension. For anxiety, the evidence suggests that these modules should include cognitive behavioural therapy (CBT) techniques that target the cognitive distortions and unhelpful behaviours underpinning the child’s anxiety. Below, we consider the evidence for each of these modules in turn. We also outline the evidence-based interventions that we selected to represent each module in the PRAX intervention used in this study.

Phonological decoding is the ability to read words using GPCs. Current evidence suggests that phonological decoding responds reliably to ‘phonics’ training, which teaches readers how to apply GPC rules during reading and/or phoneme-grapheme correspondence (PGC) rules during spelling. Meta-analyses have reported that phonics training has statistically significant moderate-to-large effects on word reading accuracy in poor readers for real words (*d* = 0.51–0.67) and for nonsense words (nonwords; *d* = 0.60–0.76; Ehri et al., 2001; McArthur et al., 2018). In the PRAX intervention used in this study, we use the Macquarie University Reading Clinic (MURC) Reading Gaps and Spelling Gaps programmes for the phonological decoding module of the PRAX schema (see “Methods” below; Kohnen & Banales, 2015a, 2015b; Kohnen, Banales & McArthur, 2020), as this intervention adopts an individualised and tailored approach to teaching children phonics.

Visual word recognition is the ability to recognise a whole written word from ‘orthographic memory’ (i.e. the written-word lexicon). This ability is often trained by reading and spelling whole words (Colenbrander et al., 2020). The success of this approach, which was initially demonstrated in case intervention studies (Broom & Doctor, 1995; Brunsdon, Coltheart & Nickels, 2005; Rowse & Wilshire, 2007), is also supported by group-controlled trials that have reported large training effects in groups of children with poor word reading ability (*d* = 1.05–1.60; McArthur et al., 2015a, 2015b). In the PRAX intervention used in this study, we used the MURC Sight Word Reading and Sight Word Spelling programmes for the visual word recognition module of the PRAX intervention schema (Kohnen & Banales, 2015c; Kohnen, Banales & McArthur, 2020), as this intervention uses a tailored and individualised flashcard approach to increasing children’s sight word knowledge.

Reading fluency is the ability read with the appropriate level of accuracy, and at the appropriate rate, for a reader’s age or grade (Wolf & Katzir-Cohen, 2001). The evidence base for reading fluency is more limited than phonological recoding or visual word recognition, nevertheless, a review by Begeny et al. (2010) of four meta-analyses and two systematic reviews identified eight potentially effective strategies for improving reading fluency. These were: reading silently whilst a skilled reader reads aloud; systematic error correction; practising a particular text; combined performance and graphical feedback; systematic praise and reward; explicit instruction to read for fluency; explicit instruction to read for comprehension; and repeated reading aloud of level-appropriate texts. Another promising technique for improving fluency is ‘wide reading’, which provides children with a variety of texts to improve the fluency of their phonological decoding and visual word recognition skills (Ardoin et al., 2016). In the PRAX intervention used in this study, we used the MURC Text Reading programme, which combines wide reading and systematic error correction, to represent the reading fluency module of the PRAX schema (Kohnen & Banales, 2015d; Kohnen, Banales & McArthur, 2020).

Once a child can read fluently, one would hope that they could understand the meaning of a text—the ultimate goal of reading. However, some children need specific help for their reading comprehension. Randomised controlled trials and meta-analyses suggest that reading comprehension intervention should include strategy instruction to allow a deep and rich interaction with text, such as question generation, activating background knowledge, and visualisation (Clarke et al., 2010; Elleman, 2017; Johnson-Glenberg, 2000). The explicit teaching of vocabulary within a text has also been effective in improving reading (Wright & Cervetti, 2017). In the PRAX intervention used in this study, we use the MURC Comprehension programme, which combines strategy instruction with vocabulary training, to represent the reading comprehension module of the PRAX intervention schema (Macquarie University Reading Clinic, 2016).

Shifting our focus to anxiety interventions, multiple systematic reviews and meta-analyses have reported that Cognitive Behavioural Therapy (CBT) is an effective psychological treatment for childhood anxiety (Kendall, 2000), with statistically significant effects for self-reported (Cohen’s *d* = 0.21–2.53), parent-reported (*d* = 0.15–3.98), and clinician-reported anxiety symptoms (*d* = 0.38–1.46). High anxiety remission rates have also been found (Cartwright-Hatton et al., 2004; Compton et al., 2002; Ginsburg et al., 2011; Ishikawa et al., 2007; James et al., 2013). In Australia, one of the most widely used CBT programmes for childhood anxiety is the Cool Kids for Anxiety programme (Lyneham et al., 2003). This programme is designed for children aged from 7 to 17 years and includes 10 weekly or fortnightly sessions of 60–90 min. Sessions can be conducted with individuals or small groups and includes parent participation. Cool Kids provides a suite of *core* modules (i.e. psychoeducation, cognitive restructuring, gradual exposures, parent management strategies) that are tailored to children’s anxiety, and additional modules (i.e. dealing with bullying, problem solving, social anxiety training, progressive muscle relaxation, social skills and assertiveness training) that focus on more specific needs.

Numerous randomised controlled trials support the efficacy of Cool Kids, reporting significantly higher remission rates for primary anxiety disorders than control treatment in various clinical, school, and research settings (Hudson et al., 2009; Chavira et al., 2014; Mifsud & Rapee, 2006; Rapee, Abbott & Lyneham, 2006). This evidence suggests that Cool Kids is an effective treatment for childhood anxiety, and hence should be incorporated into PRAX intervention. However, Cool Kids delivers its modules via written workbooks, which children with poor reading struggle to read. Thus, the anxiety modules in the PRAX intervention used Cool Kids modules that were modified for poor readers (see “Appendix A” for a detailed description of these modifications).

In summary, there is evidence that some children experience difficulties with both reading and anxiety. However, we lack an integrated intervention for children with reading and anxiety problems. Aim 1 of this study was to develop an evidence-based schema for a PRAX intervention. For reasons outlined above, this schema included four modules for reading (i.e. phonological decoding, visual word recognition, reading fluency, reading comprehension) and five modules for anxiety (i.e. social anxiety, generalised anxiety, separation anxiety, specific phobias, obsessive-compulsive symptoms). Aim 2 was to use this schema to build our own PRAX intervention. For the reading modules, we selected the MURC Reading Gaps and Spelling Gaps programmes (phonological decoding), the MURC Sight Word Reading and Sight Word Spelling programmes (visual word recognition), the MURC Text Reading programme (reading fluency) and the MURC Comprehension Scaffolding Programme (reading comprehension). For the anxiety modules, we used a modified version of Cool Kids for poor readers, which we called ‘Cool Reading’ (see “Appendix A”). Aim 3 of this study was to test the efficacy of our PRAX intervention using a methodology that was sensitive to intervention effects in individuals and the group. We therefore used WEST analyses (Howard, Best & Nickels, 2015) to assess the reading/spelling and anxiety outcomes of a double-baseline intervention case series conducted with children with comorbid reading and anxiety problems. From the existing evidence, we predicted larger gains across the intervention period than the double-baseline control period for the reading/spelling and anxiety outcomes that were directly targeted by the PRAX intervention. Given the unique nature of the PRAX intervention, we could not predict if these gains would reach statistical significance (i.e. reliability) in a 12-week period.

## Methods

### Research design

The methods for this study were approved by the Macquarie University Human Ethics committee (Reference: 5201500286). This study comprised five stages for each participant. At Test 1 (T1), children and their parents were administered screening measures and outcome measures. After a 12-week period of no training, children completed the outcome measures again at Test 2 (T2). After a 12-week PRAX intervention period, children completed the outcome measures again at Test 3 (T3). The difference in outcome scores from T1 to T2 (i.e. the double-baseline control period) was used represent non-intervention effects such as maturation and test-retest effects. The difference in outcome scores from T2 to T3 (intervention period) represented intervention effects in addition to these effects. DF (the first author) administered all tests at all time points, except for the ADIS-C/P which was administered by LM (fourth author) at T3 to reduce response bias on the more subjective anxiety outcome measure. Both clinicians received clinical supervision from JH, the second author, who is a professor of clinical psychology and an expert in childhood anxiety.

### Participants

Children were recruited from the Macquarie University Reading Clinic (MURC) and a research participant pool. No participant was receiving treatment for their reading or anxiety at T1, or between T1 and T2. Information and consent forms were sent to 11 families. Parents and children provided written and verbal consent, respectively. Eleven children and their parents were assessed at T1. Two children did not meet inclusion criteria (see below), and one parent withdrew from the study prior to T2. One participant withdrew from the study after T2. Thus, seven children completed all testing and treatment components of the study.

Children were aged between 8 and 12 years. To be included in the study, they had (1) poor reading accuracy as defined by a score at least one standard deviation (1 SD) below the age mean on the nonword reading list or irregular word reading list of the Castles and Coltheart Reading Test, 2nd Edition (CC2; Castles et al., 2009); (2) poor nonword spelling as defined by a score at least 1 SD below the age mean on the Queensland Inventory of Literacy (QUIL; Dodd et al., 1996), and (3) anxiety, as defined by a clinician severity rating of at least 4 for one or more anxiety disorders on the Anxiety Disorders Interview Schedule for Children and Parents (ADIS-C/P; Silverman & Albano, 1996; see ‘Screening measures’ for these assessments).

There was no evidence of severe behavioural issues, poor hearing, poor vision, neurological impairment, sensory impairment, or comorbid diagnoses in our sample (e.g. autism spectrum disorder, oppositional defiance disorder)—as indexed by a background questionnaire that parents completed, which was designed for the purposes of this study. There were also no significant or severe emotional problems other than anxiety (e.g. depression), as measured using the ADIS-C/P (Silverman & Albano, 1996). All children’s non-verbal IQs were within the average range (Kaufman Brief Intelligence Test 2nd Edition [KBIT-2]; Kaufman & Kaufman, 2004), as was their performance on an expressive vocabulary test of language (Assessment of Comprehension and Expression 6-11; ACE; Adams et al., 2001) for all bar three children (see “Appendix B”). The latter outcome was expected since one-third of children with poor reading struggle with various aspects of language acquisition (McArthur et al., 2000).

### Screening measures (T1)

#### Nonword and irregular word reading accuracy

The CC2 comprises three lists of 40 words that are either irregular (e.g. yacht), regular (e.g. ship), or nonwords (e.g. gop; Castles et al., 2009). Words are presented on cards in pseudo-random order of increasing difficulty. A word list is discontinued after five consecutive errors in that list. The total number of correct responses for each list can be converted into *z*-scores (*M* = 0, SD = 1). Z-scores that fall below −1, between −1 and 1, and above 1 represent performance below, within, or above the average range, respectively. We identified poor reading performance using scores one or more standard deviations below the mean (for age or grade) because we not only wanted to identify children who already had severe reading problems, but also children who were at risk of developing such problems if they were not provided with immediate support. The CC2 has sound internal consistency for irregular word and nonwords (α = 0.86 and 0.94) and good test-retest reliability irregular words and nonwords (*r* = 0.94 and 0.80; Moore et al., 2012).

#### Nonword spelling

The QUIL Nonword Spelling subtest includes 24 nonwords of increasing difficulty (e.g. dorf). Children in Grades K to 2 are administered items 1 to 12, while children above Grade 2 complete all items. Correct responses are tallied to produce a raw score out of 24. The test-retest reliability is moderately strong (*r*_*s*_ = 0.63; Dodd et al., 1996).

#### Anxiety disorders

We used the ADIS-C/P for the 4th edition of the DSM (DSM-IV) to ascertain whether a child met diagnostic criteria for one or more anxiety disorders (Silverman & Albano, 1996). In separate interviews, a child and their parent respond to a series of anxiety questions asked by a clinician. The interview per se did not increase signs of anxiety in children. If responses meet criteria for an anxiety disorder, then children and parents provide an interference rating of the anxiety. The clinician then assigned a severity rating (CSR; 0–8) to that disorder. The ADIS-C/P has sound inter-rater agreement across the major anxiety disorders (kappa = 0.68–0.93; Lyneham, Abbott & Rapee, 2007).

#### Nonverbal IQ

We used the matrices subtest of the KBIT-2 (Kaufman & Kaufman, 2004) to measure nonverbal IQ. Children pointed to the matching picture in a pattern until they made four consecutive errors. Correct responses are tallied out of a total of 46, and these scores are converted into standard scores (*M* = 100, SD = 15). Standard scores that fall below 85, between 85 and 100, or above 115 are interpreted as below, within, or above the average range, respectively. This test has good concurrent validity with the Wechsler Abbreviated Scale of Intelligence (*r* = 0.91; Kaufman & Kaufman, 2004).

#### Expressive language

We used the picture naming subtest from the Assessment of Comprehension and Expression 6-11 (ACE; Adams et al., 2001). Children are presented with 25 pictures and instructed to name the object in each picture. Correct responses are tallied and converted into scaled scores (*M* = 10, SD = 3). Scaled scores that fall below 7, between 8 and 12, or above 13 are interpreted as below, within, or above the average range. The psychometric properties of this test are sound, with reliable test-retest correlations reported (*r* = 0.83; Adams et al., 2001).

### Reading and spelling outcomes (T1, T2, and T3)

#### Accuracy

##### Individual GPCs

The Letter-Sound Test (LeST; Larsen et al., 2015) measures knowledge for individual GPCs, with norms available for children in Grades K to 3. Children are asked to pronounce the sounds of 51 individual graphemes. Correct responses are tallied to produce a raw score out of 51. The test-retest reliability for this test is sound (*r* = 0.84; Larsen et al., 2015).

##### GPCs in nonwords

The Diagnostic Reading Test (DiRT; Colenbrander, Kohnen & Nickels, 2011) was designed to assess the development of individual GPCs when read within nonwords. The shortened version used in this study includes 61 nonwords (e.g. coom) that are presented on flashcards that children read aloud. Correct responses are tallied to produce a raw score out of 61.

##### PGCs in nonwords

The Diagnostic Spelling Test for Nonwords (DiSTn; Kohnen et al., 2015) was designed to assess the development of individual PGCs. The shortened version used in this study includes 46 nonwords (e.g. mip) that children are asked to spell. Correct responses are tallied to produce a raw score out of 46.

##### Sight word reading

The Sight Word Reading Accuracy Test (Kohnen & Banales, 2015c) was used in this study to test reading accuracy of the most commonly occurring irregular words presented in order of increasing written frequency. Children are asked to read aloud words that are printed on flashcards. Testing is discontinued after 30 incorrect irregular word responses. These 30 incorrect irregular words were used in the Sight Word Reading intervention module and were used as the Sight Word Reading outcome at T2 and T3.

##### Sight word spelling

The Sight Word Spelling Test (Kohnen & Banales, 2015c) was used in this study to test spelling accuracy of the most commonly occurring irregular words that are presented in order in spelling frequency. Children are asked to spell each word that the examiner reads aloud. Testing is discontinued after a child makes 30 incorrect irregular word responses. These 30 incorrect irregular words were used in the Sight Word Spelling intervention module, and the Sight Word Spelling outcome at T2 and T3.

#### Fluency

##### Nonword reading fluency

The Phonemic Decoding subtest of the Test of Word Reading Efficiency (TOWRE; Torgesen, Wagner & Rashotte, 1999) comprises 63 nonwords (e.g. pim) that children are asked to read as quickly and accurately as possible within 45 seconds. Responses are marked as correct ‘1’ or incorrect ‘0’, tallied, and converted into standard scores (*M* = 100, SD = 15), which indicate below (<85), within (85–115) or above (>115) average performance. This test has Australian normative data (Marinus, Kohnen & McArthur, 2013), and has sound construct and criterion validity (α = > 0.90; Torgesen, Wagner & Rashotte, 1999).

##### Word reading fluency

We measured word reading fluency using the Sight Word subtest of the TOWRE (Torgesen, Wagner & Rashotte, 1999). This subtest includes 104 regular and irregular words (e.g. book) that are administered and scored using the same procedure as the Phonemic Decoding subtest (see above).

#### Comprehension

The Neale Analysis of Reading Ability 3rd edition (NARA-3; Neale, 1999) measures text reading accuracy and comprehension. It includes six increasingly complex passages that children are asked to read aloud. Their word reading errors are corrected during their reading, and they are asked to answer a series of comprehension questions at the end of each passage. Basal and ceiling passages are those in which a child makes 0–2 and 16+ word reading accuracy errors, respectively. Correctly answered questions for each passage are tallied out of a maximum score of 44. The test-retest estimate is sound (*r* = 0.93; Neale, 1999).

### Anxiety outcomes (T1, T2, and T3)

#### Disorders

Anxiety disorders were assessed using the ADIS-C/P (Silverman & Albano, 1996), which is outlined above under ‘Screening measures’. Unlike the objective outcome measure for reading and spelling, the ADIS-C/P required a degree of ‘subjective’ clinical interpretation. Thus, different clinicians were used to administer and interpret the ADIS-C/P outcomes before (DF) and after (LM) the PRAX intervention.

#### Symptoms

The Spence Children’s Anxiety Scale (SCAS-P; Nauta et al., 2004) comprises 38 items, divided into six subscales, which assess generalised anxiety, separation anxiety, social anxiety, panic/agoraphobia, physical injury fears, and obsessive-compulsive symptoms. Parents are asked to rate how often their child experiences the symptom expressed by each item (0 = never, 1 = sometimes, 2 = often, 3 = always). Scores are tallied to produce raw score for each subscale, which are converted into *T*-scores (*M* = 50, SD = 10). *T*-scores above 60 are considered ‘elevated’ based on normative data from large Australian and Dutch community sample (*N* = 745; children aged 6–18 years: *M* = 10.80 years, SD = 2.40 years; *n* = 389 boys, *n* = 356 girls; Nauta et al., 2004). The internal consistency for the subscales is sound (α = 0.61–0.81), as is the discriminant validity (Wilks lambda 0.65, *p* < 0.001), and test-retest reliability (*r* = 0.53 to 0.85).

### Intervention

The intervention modules used in the PRAX intervention are summarised in Tables 1 and 2. These tables also include the selection criteria and tests we used to decide if a module should be included in the PRAX intervention.

**Table 1:** The reading modules (first column), selected interventions (second column), and selection criteria (third column) used in the PRAX intervention in the current study.

Reading modules	Selected interventions	Selection criteria	
Accuracy			
GPCs	MURC Reading Gaps	< -1 SD CC2 Nonwords	
PGCs	MURC Spelling Gaps	< -1 SD QUIL	
Sight Word Reading	MURC Sight Word Reading	< -1 SD CC2 Irregular Words	
Sight Word Spelling	MURC Sight Word Spelling	< -1 SD DiSTi	
Fluency	MURC Text Reading	< -1 SD TOWRE Sight Words	
Comprehension	MURC Comprehension	< -1 SD NARA-3	

**Note:**

< -1 SD indicates more than one standard deviation below the mean expected for age or grade.

**Table 2:** The core and additional anxiety modules (first column), brief module descriptions (fourth column), and selection criteria (final column) used in the PRAX intervention in the current study.

Anxiety modules	Core	Additional	Description	Selection criteria
Psychoeducation	X		Teach children about the experience of anxiety, as well as prognosis, and outcomes associated with intervention.	Clinician Severity Rating ≥ 4 ADIS-C/P
Cognitive Restructuring	X		Teach children strategies to challenge maladaptive or unhelpful anxious thoughts	
Gradual Exposure	X		Teach children strategies to face their fears through new learning experiences that are tailored to their specific worries	
Child management strategies for parents	X		Teach parents strategies to help their children cope with anxiety	
Social skills and confidence		X	Teach children basic social skills	Parent/child/clinician report: social difficulties, passive behaviour, aggressive behaviour
Structured problem solving		X	Teach children strategies to identify and solve problems	Parent/child/clinician report: Difficulty defining worries, or brainstorming solutions, or selecting solutions.
Dealing with bullying		X	Teach children strategies to cope with bullies	Parent/child/clinician report: Child is being bullied which is contributing to their anxiety
Progressive muscle relaxation		X	Teach children relaxation strategies	Parent/child/clinician report: Elevated physiological symptoms, such as inability to sleep or rapid breathing during treatment
Social anxiety training		X	Teach children specific strategies to reduce social anxiety (e.g., attention training and safety behaviour gradual exposures)	Parent/child/clinician report: Severe social anxiety

The reading modules were administered in the order that follow how the skills are typically mastered; that is accuracy followed by fluency and then comprehension (Adams, 1990; Golinkoff, 1975; Laberge & Samuels, 1974). If a child had a problem with all these skills, they started with modules for accuracy then move to fluency and then to comprehension. All children were administered accuracy and fluency modules (see descriptions below) but did not graduate to comprehension modules.

For anxiety, each child was administered the core Cool Reading modules—carefully tailored to their individual anxiety—in the order outlined in Table 2. Children with related anxiety problems were administered relevant additional Cool Reading modules. Core and additional anxiety modules that were administered to children in this study are outlined below.

The PRAX intervention was administered in three 1-h sessions per week for 12 weeks (36 h of training in total). It comprised 21.75 h of reading modules and 14.25 h of anxiety modules. The time spent on each module in each session for each child are shown in Table 3. The total treatment was equivalent to 10 weeks of reading and spelling treatment at the MURC (i.e. three 45-min sessions per week for 10 weeks) and 10 weeks of anxiety treatment in Cool Kids (i.e. one 60-to-90-min session per week for 10 weeks). Treatment was delivered to children in person at the MURC or online using Zoom. Research suggests that in-person and online interventions for reading/spelling and anxiety are similarly effective (Bouchard et al., 2004; Kohnen, Banales & McArthur, 2020).

**Table 3:** Interventions administered in each session of our PRAX intervention. Shaded cells indicate a 1-h in-session gradual exposure completed with the clinician, parent, and child.

Week	Session	Reading Modules						Anxiety modules						
		Reading gaps	Spelling gaps	Sight word reading	Sight word spelling	Text reading	Duration (minutes)	Psychoeducation	Controlled breathing	Cognitive restructuring	Gradual exposure	Parent strategies	Parent check in	Duration (minutes)
1	1						0	✓				✓		60
	2						0	✓	✓	✓		✓		60
	3	✓	✓	✓	✓	✓	45		✓	✓			✓	15
2	4	✓	✓	✓	✓	✓	45		✓	✓			✓	15
	5	✓	✓	✓	✓	✓	45		✓	✓			✓	15
	6	✓	✓	✓	✓	✓	45		✓			✓		15
3	7	✓	✓	✓	✓	✓	45		✓	✓			✓	15
	8	✓	✓	✓	✓	✓	45		✓		✓		✓	15
	9	✓	✓	✓	✓	✓	45		✓			✓		15
4	10	✓	✓	✓	✓	✓	45		✓		✓		✓	15
	11	✓	✓	✓	✓	✓	45		✓		✓		✓	15
	12	✓	✓	✓	✓	✓	45		✓			✓		15
5	13	✓	✓	✓	✓	✓	45		✓		✓	✓		15
	14	✓	✓	✓	✓	✓	45		✓		✓		✓	15
	15	✓	✓	✓	✓	✓	45		✓		✓		✓	15
6	16	✓	✓	✓	✓	✓	45		✓	✓			✓	15
	17	✓	✓	✓	✓	✓	45		✓		✓		✓	15
	18	✓	✓	✓	✓	✓	45		✓		✓	✓		15
7	19	✓	✓	✓	✓	✓	45		✓			✓		15
	20	✓	✓	✓	✓	✓	45		✓		✓		✓	15
	21	✓	✓	✓	✓	✓	45		✓		✓			15
8	22	✓	✓	✓	✓	✓	45		✓				✓	15
	23	✓	✓	✓	✓	✓	45		✓		✓		✓	15
	24						0				✓			60
9	25	✓	✓	✓	✓	✓	45		✓				✓	15
	26	✓	✓	✓	✓	✓	45		✓		✓		✓	15
	27						0				✓		✓	60
10	28	✓	✓	✓	✓	✓	45		✓		✓		✓	15
	29	✓	✓	✓	✓	✓	45		✓	✓	✓	✓		15
	30						0				✓		✓	60
11	31	✓	✓	✓	✓	✓	45		✓				✓	15
	32	✓	✓	✓	✓	✓	45		✓		✓		✓	15
	33						0				✓		✓	60
12	34	✓	✓	✓	✓	✓	45		✓	–Coping		✓		15
	35	✓	✓	✓	✓	✓	45		✓	–Helping			✓	15
	36						0	–Goal and progress review –Plan for the future –Certificate						60
	Reading treatment time						21.75	Anxiety treatment time						14.25

#### Reading accuracy modules

##### MURC Reading Gaps (Kohnen & Banales, 2015a)

This module trained each child to read the GPCs that they failed to read correctly during the DiRT and LeST assessments at T1. GPCs were introduced in order of frequency of occurrence in English. Two GPCs were introduced per session, which were typically mastered (100% accuracy in two consecutive sessions) in one to six sessions. For each GPC, the child was shown the grapheme and told the sound. The child pronounced and blended each GPC within 10–12 words and/or nonwords that comprise already-acquired GPCs. Immediate feedback was provided for all attempts.

##### MURC Spelling Gaps (Kohnen & Banales, 2015b)

This module used spelling to train PGCs, which is an effective intervention for word reading accuracy in poor readers (Ouellette, Martin-Chang & Rossi, 2017). Target PGCs for each child were identified from their errors in the DiSTn at T1. Each PGC was trained using the same procedure as the MURC Reading Gaps (see above) except words were spelled rather than read.

##### MURC Sight Word Reading (Kohnen & Banales, 2015c)

This module trained the first 30 incorrect words read by each child during the Sight Word Reading Accuracy Test at T1. Four to eight words were trained per session. Children were asked to read each word, copy the word, say the letter names, visualise the spellings, spell the word, focus on the irregular parts of the spelling, and silently rehearse the letters in the word in their mind. Children were given immediate feedback on their performance. Typically, children mastered a word in one to six sessions.

##### MURC Sight Word Spelling (Kohnen & Banales, 2015c)

This module trained the first 30 words spelled incorrectly by a child on the Sight Word Spelling Accuracy Test at Test 1. The procedure was the same as the MURC Sight Word Reading (see above) except that children spelled rather than read the words.

#### Reading fluency modules

##### MURC Text Reading.

In this module, children read phonics readers focussing on the GPCs practiced in Reading Gaps, or ‘real’ books which have been selected so children read with 90% accuracy. Children read aloud while the clinician follows along silently and corrects any incorrect words. Correction procedures differ depending on the child’s mistake. Clinicians may prompt their phonics knowledge, ask the children to slightly adjust a sounding-out attempt to pronounce an irregular word correctly (Savage et al., 2018), or prompt to break up longer words. Children are taught ‘careful reading’ techniques, which encourages them to self-correct their reading errors and teaches them to use their finger to track letters and words in the sentences.

#### Core anxiety modules

##### Psychoeducation

This module educated parents and children about anxiety and explained the rationale for CBT. Through a series of cartoon videos, children learned about the experience of anxiety, how it might impact their body (e.g. physical symptoms), their thoughts (e.g. unhelpful thinking), and their behaviour (e.g. avoidance). They also learned about the potential causes of anxiety, and how treatment may help over time. Children learned to evaluate the intensity of their anxiety using a worry rating scale and completed activity sheets on thoughts and feelings.

##### Controlled breathing

Controlled breathing is a strategy to help children lower their physiological arousal and tolerate feelings of distress when anxious. Children watched a cartoon of a child practising controlled breathing and practiced breathing in session. They practiced controlled breathing at the start of every session, and immediately before reading, to reduce reading-related anxiety and increase engagement in reading intervention.

##### Cognitive restructuring

This module taught children to modify their unrealistic thoughts, expectations, and beliefs associated with anxiety. Cartoon videos and worksheets were used to teach children how to identify and modify anxious thoughts and use realistic thoughts to lower anxiety. Children were also taught how to use visual worksheets outside sessions to help with anxious thoughts.

##### Gradual exposure

This module gradually exposed children to the source of their anxiety to encourage new learning. They watched cartoon videos of other children completing gradual exposures and learned to develop their own gradual exposure activities in session. Gradual exposure was introduced in Week 5, once children had acquired skills in controlled breathing, cognitive restructuring, and gained confidence reading.

##### Child management strategies for parents

This module provides parents with strategies to help their child cope with anxiety. Parents were provided with information about their child’s anxiety, factors that maintained their child’s anxiety, and suggestions on how to support their child to independently cope. This training was provided fortnightly without the child.

#### Additional anxiety modules

##### Social skills and confidence

This module was selected when children had difficulties with their peers and social skills (e.g. extremely shy or exhibiting behavioural outbursts). Role plays were used in session to teach children how to improve their social interactions with their peers, as well as boost their confidence. These activities were integrated into each session as required.

##### Structured problem solving

This module was selected for children who sought excessive reassurance (e.g. relied excessively on parents to solve problems). It taught them problem-solving skills via activity sheets that focused on identifying problems, brainstorming solutions, and planning actions. Structured problem solving was integrated into sessions when difficulties were identified.

##### Dealing with bullying.

This module was selected for children being bullied. It taught children how to cope with bullying using strategies (i.e. clever comebacks to say to bullies) and role play exercises that built assertiveness skills.

##### Progressive muscle relaxation.

This module was selected for children who experienced elevated arousal that reduced their capacity to complete the core anxiety modules. It used a relaxation script to instruct children how to progressively tense and relax their muscles.

##### Social anxiety training

This module was selected for children with social anxiety. It taught children strategies to overcome anxiety related to performance or social embarrassment. It included attention training exercises (i.e. learning to focus attention), safety behaviour experiments (i.e. practice task without avoidant behaviour such as speaking with head down), and video feedback experiments (i.e. watching a video of the gradual exposure attempt to gain more evidence about the actual experience).

## Results

### Profiles of participants

Three girls (S1, S2, S3) and four boys (S4, S5, S6, S7), aged between 8 and 12 years (*M* = 8.94, SD = 0.82; Range: 8 to 12 years), met inclusion criteria for this study. Children presented with various reading and spelling difficulties (see Table 4 for reading screening outcomes). All children performed at least 1 SD below the age mean for nonword reading accuracy, irregular word reading accuracy, and nonword spelling accuracy. Four children met criteria for a primary diagnosis of social anxiety disorder (S1, S4, S5, S7), two children for generalised anxiety disorder (S3, S6), and one child for separation anxiety disorder (S2). Three children were diagnosed with separation anxiety disorder (S2, S4, S5), and six children with one or more specific phobias (S1, S2, S3, S4, S5, S7). Six children reported significant and interfering worries related to reading (S1, S2, S4, S5, S6, S7).

**Table 4:** Children’s scores at T1 on the screening measures.

	S1	S2	S3	S4	S5		S6	S7
**Reading (z scores)**^a^								
Nonword reading accuracy	−2.31	−1.06	−1.93	−2.37	−1.25		−2.29	−1.20
Irregular reading accuracy	−2.12	−2.51	−2.16	−1.87	−2.00		−1.63	−2.08
**Spelling (scaled scores)**^a^								
Nonword spelling accuracy	3	5	5	3	4		4	6
**Anxiety disorders (CSR)**^b^								
Social anxiety	6	6	ND	5		6	ND	5
Generalised anxiety	5	ND	4	4		4	4	4
Separation anxiety	ND	6	ND	4		5	ND	ND
Specific phobias	Spiders: 4 Dark: 4 Clowns: 4 Doctor: 4	Wasps: 5 Loud noises: 5 Spiders: 4 Lifts: 4 Toilets: 4	Dogs: 4 Lifts: 4 Insects: 4 Dentist:4 Dark: 4 Blood: 4 Spiders: 4	Heights: 4 Planes: 4		Dark: 4	ND	Spiders: 4 Heights: 4

**Notes:**

a Low scores = poor performance.

b High scores = poor performance.

ND, not diagnosed.

Grey cells = primary anxiety disorder.

### Reading and spelling outcomes

We evaluated the effectiveness of PRAX intervention on reading and spelling outcomes using weighted statistics (WEST; Howard, Best & Nickels, 2015). There are two key statistics of interest: WEST-Trend, which is calculated to evaluate change over the entire study period (i.e. the change in scores from T1 to T2 to T3), and WEST-ROC, which is calculated to evaluate change specific to the intervention period (i.e. T2 to T3). In this study, a PRAX intervention effect was only considered significant if both WEST-Trend (overall) and WEST-ROC (specific to the intervention) were statistically significant.

We conducted a group analysis using West-Trend and WEST-ROC to evaluate if there was a change in the raw scores at the group level for each reading outcome. We also conducted an analysis using WEST-Trend and WEST-ROC to analyse individual treatment responses for each outcome. For the individual analysis, each item on a test was assigned a dichotomous rating of correct (e.g. score of 1) or incorrect (e.g. score of 0). The child’s response on each item was compared against the same item at each time point (i.e. item 1 score at T1, T2, T3; item 2 score at T1, T2, T3 etc.). The raw score was then multiplied by a weight and subjected to a one-sample *t*-test.

The raw scores and WEST-Trend and WEST-ROC values for the reading/spelling outcome measures are shown in Table 5. This table also indicates which intervention each child received.

**Table 5:** Raw scores and WEST-ROC and WEST-TREND results for reading/spelling outcomes, with an indication of whether each skill was trained during treatment.

Outcome	Test	S1	S2	S3	S4	S5	S6	S7	M (SD)
Individual GPCs (raw/51)	T1	38	34	27	28	36	26	31	31.43 (4.69)
	T2	42	35	29	29	39	25	34	33.29 (6.02)
	T3	46	50	33	43	46	37	45	42.86 (5.87)
	WEST-Trend	*	*	ns	*	*	*	*	*t* (6) = 8.09, *p* < 0.001
	WEST-ROC	ns	*	ns	*	ns	*	*	*t* (6) = 3.52, *p* < 0.01
	Trained	✓	✓	✓	✓	✓	✓	✓	
GPCs in nonwords (raw/61)	T1	10	18	11	0	15	1	20	10.71 (7.83)
	T2	13	16	11	1	16	4	9	10.00 (5.77)
	T3	37	40	12	9	24	7	27	22.29 (13.36)
	WEST-Trend	*	*	ns	*	*	*	*	*t* (6) = 3.28, *p* < 0.05
	WEST-ROC	*	*	ns	*	ns	ns	*	*t* (6) = 2.85, *p* < 0.05
	Trained	✓	✓	✓	✓	✓	✓	✓	
PGCs in nonwords (raw/46)	T1	7	13	7	1	18	3	16	9.29 (6.50)
	T2	3	4	1	2	16	1	16	6.15 (6.82)
	T3	31	34	6	11	28	6	21	19.57 (11.93)
	WEST-Trend	*	*	ns	*	*	ns	*	*t* (6) = 2.95, *p* < 0.05
	WEST-ROC	*	*	*	*	*	*	*	*t* (6) = 3.27, *p* < 0.05
	Trained	✓	✓	✓	✓	✓	✓	✓	
Sight word spelling (raw/30)	T1	0	0	0	0	0	0	0	0.00 (0.00)
	T2	6	7	0	6	6	3	5	22.14 (6.34)
	T3	25	25	10	25	25	17	28	6.57 (3.31)
	WEST-Trend	*	*	*	*	*	*	*	*t* (6) = 9.25, *p* < 0.001
	WEST-ROC	*	*	*	*	*	*	*	*t* (6) = 12.80, *p* < 0.001
	Trained	✓	✓	✓	✓	✓	✓	✓	
Sight word reading (raw/30)	T1	0	0	0	0	0	0	0	0.00 (0.00)
	T2	17	2	7	14	14	10	15	11.29 (5.28)
	T3	27	28	24	25	27	26	28	26.43 (1.51)
	WEST-Trend	*	*	*	*	*	*	*	*t* (6) = 46.25, *p* < 0.001
	WEST-ROC	ns	*	*	ns	ns	ns	ns	*t* (6) = 0.97, *p* > 0.05
	Trained	✓	✓	✓	✓	✓	✓	✓	
Nonword reading fluency(raw/63)	T1	4	11	9	4	7	2	9	6.57 (3.31)
	T2	8	5	10	3	13	5	11	7.86 (3.67)
	T3	13	10	8	11	11	3	7	9.00 (3.32)
	WEST-Trend	*	ns	ns	*	*	ns	ns	*t* (6) = 1.49, *p >* 0.05
	WEST-ROC	ns	*	ns	*	ns	ns	ns	*t* (6) = −0.05, *p >* 0.05
	Trained	X	X	X	X	X	X	X	
Word reading fluency(raw/104)	T1	34	20	14	33	37	37	29	29.14 (8.90)
	T2	35	25	16	37	42	36	31	31.71 (8.71)
	T3	46	29	20	40	45	43	34	36.71 (9.59)
	WEST-Trend	*	*	*	*	*	ns	ns	*t* (6) = 8.45, *p* <.001
	WEST-ROC	*	ns	ns	ns	ns	ns	ns	*t* (6) = 1.36, *p >* 0.05
	Trained	✓	✓	✓	✓	✓	✓	✓	
Reading comprehension (raw/44)	T1	12	2	4	12	10	6	6	7.43 (3.95)
	T2	12	9	4	10	10	9	10	9.14 (2.48)
	T3	11	10	4	12	18	10	13	11.14 (4.18)
	WEST-Trend	ns	*	ns	ns	*	*	*	*t* (6) = 2.44, *p* < 0.05
	WEST-ROC	ns	ns	ns	ns	*	ns	ns	*t* (6) = 0.17, *p >* 0.05
	Trained	X	X	X	X	X	X	X	

**Note:**

✓ and X indicate that a child was or was not (respectively) trained in skills that are indexed by that outcome measure.

#### Accuracy

##### Individual GPCs

All children were administered the MURC Reading Gaps programme. At the group level, there was a statistically significant effect of PRAX intervention on this outcome, which was also the case for four individual children (S2, S4, S6, S7).

##### GPCs in nonwords

All children received the MURC Reading Gaps program. At the group level, there was a statistically significant effect of PRAX intervention on this outcome, which was also seen in four individual children (S1, S2, S4, S7).

##### PGCs in nonwords

All children were administered the MURC Spelling Gaps programme. There was a statistically significant PRAX intervention effect on this outcome at the group level, as well as at the individual level for five children (S1, S2, S4, S5, S7).

##### Sight word spelling

All children received the MURC Sight Word Spelling programme. At the group level, there was a statistically significant effect of PRAX intervention on this outcome, which was also the case for all seven children.

##### Sight word reading

All children were administered the MURC Sight Word Reading programme. At the group level, there was a statistically significant effect of PRAX intervention on this outcome, which was also the case for two individual children (S2, S3).

#### Reading fluency

All children received the MURC Text Reading programme. At the group level, there was no significant intervention effect for word or nonword reading fluency. Significant treatment effects were shown by one child for nonword reading fluency (S4), and one child for word reading fluency (S1).

#### Reading comprehension

As outlined above, no child received intervention for this skill. At the group level, there was no significant treatment effect for reading comprehension. At the individual level, significant treatment effects were observed for just one child (S5).

### Anxiety outcomes

We evaluated the effect of PRAX intervention on anxiety disorders and symptoms at the group level using repeated-measures ANOVAs to compare anxiety outcomes at T1, T2, and T3. Paired samples *t-*tests were used to identify if there were significant reductions in anxiety from T2 to T3 compared to T1 to T2. It is important to note that the data is not likely to be normally distributed, which means the Type I error rate may be inflated (Oberfeld & Franke, 2013). For completeness, we also analysed the data using a non-parametric Friedman’s test and report the Q statistic alongside the traditional parametric tests.

#### Disorders

The raw scores on the anxiety disorder outcome measures are shown in Table 6. Children were administered core and additional treatment modules for social anxiety (*N* = 6), generalised anxiety (*N* = 5), separation anxiety (*N* = 3), and specific phobias (*N* = 7). As a group, there was a significant reduction in the clinical severity of social anxiety disorder across intervention period but not the baseline period.

**Table 6:** Raw scores, repeated measures ANOVA, and Friedman statistics for anxiety disorder outcomes, with an indication of whether each disorder was trained during treatment.

		S1	S2	S3	S4	S5	S6	S7	Mean (SD)	Statistic
Disorders (ADIS-C/P; CSR raw scores/8)										
Social	T1	6	6	ND	5	6	ND	5	4.67 (2.34)	*F* (2, 8) = 18.96, *p* < 0.01 *Q* = 10.00, *p* = 0.007
	T2	6	6	ND	5	6	4	5	5.33 (0.82)	
	T3	4	**0**	ND	**3**	**3**	**3**	**2**	2.50 (1.38)	
	Trained	✓	✓	X	✓	✓	✓	✓		
Generalised	T1	5	ND	4	4	4	5	4	4.33 (0.52)	*F* (2, 8) = 5.16, *p* < 0.05 *Q* = 5.20, *p* = 0.07
	T2	5	ND	4	4	ND	4	4	3.50 (1.76)	
	T3	**3**	ND	**3**	**3**	ND	5	**2**	2.67 (1.63)	
	Trained	✓	X	✓	✓	X	✓	✓		
Separation	T1	ND	6	ND	4	5	ND	ND	5.00 (1.00)	*F* (2, 4) = 2.00, *p* > 0.05 *Q* = 2.00, *p* > 0.05
	T2	ND	6	ND	5	5	ND	ND	5.33 (0.58)	
	T3	ND	4	ND	5	**2**	ND	ND	3.67 (1.53)	
	Trained	X	✓	X	✓	✓	X	X		
Phobias	T1	Spiders: 4 Dark: 4 Clowns: 4 Doctor: 4	Wasps: 5 Loud noises: 5 Spiders: 4 Lifts: 4	Dogs: 4 Lifts: 4 Insects: 4 Dentist:4 Dark: 4 Blood: 4 Spiders: 4	Heights: 4 Planes: 4	Dark: 4	ND	Spiders: 4 Heights: 4		
	T2	Spiders: 4 Dark: 4 Clowns: 4 Doctor: 4	Wasps: 5 Loud noises: 5 Spiders: 4 Lifts: 4	Dogs: 4 Lifts: 4 Insects: 4 Dentist:4 Dark: 4 Blood: 4 Spiders: 4	Heights: 4 Planes: 4	Dark: 4	ND	Spiders: 4 Heights: 4		
	T3	Spiders: **0** Dark: **0** Clowns: **0** Doctor: **2**	Wasps: **2** Loud noises: **0** Spiders: 4 Lifts: **2**	Dogs: **3** Lifts: **0** Insects: **3** Dentist: 4 Dark: **0** Blood: 4 Spiders: **2**	Heights: **0** Planes: **0**	Dark: **3**	ND	Spiders: **0** Heights: **0**		

**Note:**

Scores falling below the clinical threshold at T3 are bolded. ✓ and X indicate that a child was or was not (respectively) trained in skills that are indexed by that outcome measure.

We also examined remission rates (i.e. no longer meeting diagnostic criteria) by comparing anxiety disorder scores at T1, T2, and T3 to a clinical cut-off score (CSR ≤ 4) to identify the percentage of children who shifted from above cut-off at T1 and T2 to below cut-off at T3 (see Table 7). Overall, 57% of children did not meet diagnostic criteria for their primary diagnosis after the intervention, and 42% of children were free from all anxiety disorders after the intervention. More specifically, social anxiety disorder remitted for five of six children (83%), generalised anxiety remitted for four of five children (80%), separation anxiety remitted for one of three children (33%), and at least one specific phobia remitted for six of six children (85%).

**Table 7:** Diagnostic remission rates for all participants on the outcome measure for all anxiety disorders.

Disorder	Free from diagnosis	
	*n*	%
All diagnoses	3/7	42
Primary diagnoses	4/7	57
Social anxiety disorder	5/6	83
Generalised anxiety disorder	4/5	80
Separation anxiety disorder	1/3	33
Specific phobia	17/20	85
Total	27/34	79

**Note:**

Some children received multiple specific phobia diagnoses.

#### Symptoms

At the individual level, we conducted a baseline adjusted change (BAC) score ([T3-T2]–[T2-T1]) and compared this score to a critical value—calculated from the test-retest reliability estimate and the SD of the normative sample—to determine if there was a significant intervention effect using one-tailed *t*-tests with an alpha level of 0.05 (see “Appendix C” for details of this approach).

Raw scores, statistics, and intervention details for anxiety symptom outcomes are shown in Table 8. As a group, there were significant reductions for total, social, generalised, and separation anxiety symptoms over the intervention period. There was also a significant reduction for panic/agoraphobia symptoms. Individually, five children showed significant reductions for total anxiety (S1, S2, S4, S5, S6), four children showed significant reductions for social (S1, S4, S5, S6) and generalised anxiety (S2, S4, S5, S6), three children showed significant reductions for separation anxiety (S1, S2, S6), and single showed reductions for children for physical injury fears (S3), obsessive compulsive symptoms (S1), and panic/agoraphobia symptoms (S4).

**Table 8:** Raw scores and repeated measures ANOVA statistics for parent-reported anxiety symptom outcomes, with an indication of whether each symptom was trained during treatment.

		S1	S2	S3	S4	S5	S6	S7	Mean (SD)	Statistic
Social anxiety (SCAS-P; raw scores)	T1	16	11	5	11	7	7	8	**9.28 (3.68)**^**a**^ **10.14 (4.84)**^**a**^ **5.00 (2.71)**^**b**^	*F* (2, 12) = 8.97, *p* < 0.01
	T2	17	7	2	13	10	13	9		*Q* = 7.63, *p* = 0.022
	T3	8	4	1	5	4	4	3		
	BAC (+/− 5.1)	**−10**	1	2	**−10**	**−9**	**−15**	−1		
Trained		✓	✓	X	✓	✓	✓	✓		
Generalised anxiety (SCAS-P; raw scores)	T1	7	5	7	11	6	5	3	**6.28 (2.49)**^**a**^	*F* (2, 12) = 9.89, *p* < 0.05
	T2	7	11	6	13	8	8	2	**7.85 (3.53)**^**a**^	*Q* = 7.15, *p* = 0.028
	T3	5	3	3	7	4	2	3	**3.85 (1.67)**^**b**^	
	BAC (+/- 5.0)	−2	**−14**	−2	**−8**	**−6**	**−9**	2		
Trained		✓	X	✓	✓	X	✓	✓		
Separation anxiety (SCAS-P; raw scores)	T1	8	13	9	14	8	11	6	**9.85 (2.91)**^**a**^	*F* (2, 12) = 24.43, *p* < 0.001
	T2	10	16	7	12	10	12	5	**10.28 (3.59)**^**a**^	*Q* = 10.57, *p* = 0.005
	T3	4	9	1	7	7	5	3	**5.14 (2.73)**^**b**^	
	BAC (+/− 5.6)	**−8**	**−10**	−4	−3	−5	**−8**	−1		
Trained		X	✓	X	✓	✓	X	X		
Physical injury fears (SCAS-P; raw scores)	T1	8	4	14	2	4	3	3	5.42 (4.23)	*F* (2, 12) = 2.04, *p >* 0.05
	T2	7	4	14	2	4	4	3	5.42 (4.07)	*Q* = 0.63, *p* > 0.05
	T3	4	2	8	3	1	4	5	3.85 (2.26)	
	BAC (+/− 4.4)	−2	−2	**−6**	1	−3	−1	2		
Trained		X	X	X	X	X	X	X		
Obsessive compulsive (SCAS-P; raw scores)	T1	5	0	1	4	1	0	2	1.85 (1.95)	*F* (2, 12) = 3.78, *p* > 0.05
	T2	6	0	1	2	2	1	2	2.00 (1.91)	*Q* = 7.90, *p* = 0.019
	T3	0	0	0	2	0	0	1	0.42 (0.78)	
	BAC (+/− 4.1)	**−7**	0	−1	2	−3	−2	−1		
Trained		X	X	X	X	X	X	X		
Panic/agoraphobia (SCAS-P; raw scores)	T1	3	2	3	4	0	3	0	**2.14 (1.57)**^**a**^	*F* (2, 12) = 4.58, *p* < 0.05
	T2	3	1	3	7	0	2	0	**2.28 (2.42)**^**a**^	*Q* = 6.12, *p* = 0.047
	T3	0	1	1	4	0	0	0	**0.85 (1.46)**^**a**^	
	BAC (+/− 4.4)	−3	1	−2	**−6**	0	−1	0		
Trained		X	X	X	X	X	X	X		
Total anxiety (SCAS-P; raw scores)	T1	47	35	39	46	26	29	22	**34.85 (9.71)**^**a**^	*F (*2, 12) = 22.58, *p* < 0.001
	T2	50	39	33	49	34	40	21	**38.00 (10.00)**^**a**^	*Q* = 9.85, *p* = 0.007
	T3	21	19	14	28	16	15	21	**19.14 (4.81)**^**b**^	
	BAC (+/− 20.4)	**−32**	**−24**	−13	**−24**	**−26**	**−36**	1		
Trained		✓	✓	✓	✓	✓	✓	✓		

**Notes:**

Significant effects (*p* < 0.05) are bolded.

Non-matching superscripts indicate significant differences.

✓ and X indicate that a child was or was not (respectively) trained in skills that are indexed by that outcome measure.

Grey cells indicate the symptom measure associated with the primary diagnosis for each child.

## Discussion

The aims of the current study were to measure the effects of the first PRAX intervention in children with comorbid reading and anxiety problems. We predicted larger gains across the intervention period than the double baseline control period for outcomes that were directly targeted by the PRAX intervention. The unique nature of the intervention proscribed predictions of whether such gains would reach statistical significance in a 12-week period.

### Reading outcomes

In this study, all children received programmes that aimed to improve their reading accuracy by training their GPCs, PGCs, sight word reading, or sight word spelling. At the group level, all these skills showed reliable intervention effects except sight word reading, which showed a significant WEST-Trend but not WEST-ROC. The same pattern of results was seen at the individual level: the majority of children made significant improvements in all these skills except sight word reading. Considered together, these group and individual outcomes suggest that the PRAX intervention had a significant impact on most of the skills that were targeted by programmes to improve reading accuracy.

The limited effect of PRAX intervention at the individual level for sight word reading (i.e. only two children) was somewhat puzzling given that we have found large and significant effects of reading intervention on these skills in at least two previous studies with children with developmental dyslexia (McArthur et al., 2015a; 2015b). This could be explained by the large test-retest effects on the sight word reading test, which were one-third of the size of the (large) gains over the intervention period (T2 to T3). It is possible that the orthographic representations for a large proportion of the 30 words selected for sight word training at T1 were ‘teetering’ at a threshold that allowed them to be read correctly at T2. Although PRAX intervention period doubled those gains again, this ratio was not great enough to satisfy the algorithm generating the WEST-ROC statistic.

Our PRAX intervention programme also delivered all children with an intervention aimed to increase reading fluency. However, reliable intervention effects were only observed in two of the seven children, one who improved in nonword reading fluency, and one in word reading fluency. There are a number of possible explanations for these limited effects. First, the reading fluency outcome, which asked children to read lists of words, was not well matched to the intervention, which asked children to read words in texts. Second, while the reading accuracy of children improved significantly in this study, the severity of their reading difficulties meant that their reading abilities remained poor. It was also observed during the reading fluency intervention module that children continued to read slowly and accurately, rather than gain speed and confidence. This observation relates to third intriguing explanation. The first author has noted, when working with numerous children with PRAX, that many adopt an unusual speed-accuracy trade-off. Specifically, they tend to read ahead before reading aloud to minimise their chance of making mistakes in front of other people. This observation, which we aim to investigate empirically as soon as is feasible, offers another explanation for why children did not benefit from the PRAX reading fluency module in this study.

Regarding reading comprehension, no child mastered the reading accuracy and fluency modules and hence none received specific reading comprehension intervention. This is one explanation for why there was no significant effect of PRAX intervention at the group level, and why only one child showed significant improvements at the individual level. A second explanation is that poor reading comprehension is quite difficult to improve. Previous studies that have administered reading comprehension interventions to children with poor reading have met with mixed or limited success, especially when using standardised rather than bespoke outcome measures (Rogde et al., 2019; Wright & Cervetti, 2017). At this point in time, it would appear that poor reading comprehension is a highly complex cognitive problem that we do not fully understand, and hence do not yet know how to treat in a reliable way.

### Anxiety outcomes

The effect of the PRAX intervention on anxiety was also encouraging. The results showed that 79% of children’s anxiety disorders were no longer present after intervention, and there was a promising remission rate of 57% for primary anxiety disorders. In addition, there were statistically significant reductions for social anxiety disorder, as well as numerous anxiety symptoms (social anxiety, generalised anxiety, separation anxiety, panic/agoraphobia) over the intervention period. These results suggest that the PRAX intervention had a positive and significant effect to reduce anxiety disorders and symptoms in poor readers with anxiety.

It is important to acknowledge the slightly lower (i.e. 57%) remission rate compared to other Cool Kids studies (68%; Hudson et al., 2009), particularly given the frequency and intensity of sessions (i.e. 3 sessions per week) and dose of gradual exposures (i.e. six sessions). One explanation may be the complex nature the children’s presenting problems. Six children had severe reading anxiety, and pervasive perfectionistic tendencies, fear of embarrassment, and school avoidance. Thus, it is possible that a larger ‘dose’ of anxiety intervention may be required to further improve overall remission rates for children with comorbid reading and anxiety difficulties. In addition, the youngest child also had comorbid language difficulties and did not remit from their primary diagnosis. Thus, being younger and experiencing language difficulties may contribute to poorer outcomes in the current PRAX intervention, which necessitates further modifications to optimise outcomes for all children.

### Clinical implications

This study found that PRAX intervention had a significant and positive effect on children’s poor reading and spelling accuracy, as well as their anxiety disorders and symptoms. This is an important finding and provides the first evidence for an intervention to concurrently improve reading and anxiety for children with these comorbid difficulties. This is a crucial first step in supporting children with these difficulties given the huge burden that poor reading and anxiety place on a child’s life. These findings justify a randomised controlled trial to further assess the size and reliability of PRAX intervention effect (NHMRC, 2000). Such a trial will take time to conduct and publish. We therefore provide the following recommendations for clinicians who wish to administer their own PRAX intervention to children with poor reading and anxiety.

The first recommendation is to provide a comprehensive assessment of children’s reading and anxiety problems, and then identify effective interventions that match their exact needs. This requires the identification of evidence-based assessments and interventions for reading/spelling accuracy, reading fluency, and reading comprehension, as well social anxiety, separation anxiety, specific phobias, generalised anxiety. Tables 1 and 2 provide some options for appropriate assessments and intervention modules.

Once a suite of evidence-based intervention modules has been identified, we suggest that they are integrated into intervention sessions in the following way. First, sessions 1 and 2 should focus on anxiety. Session 1 should provide introductions, goal setting, and psychoeducation, and Session 2 should introduce cognitive restructuring and a relaxation strategy. In Sessions 3 to 7, reading and anxiety interventions should be combined by starting with a relaxation strategy immediately before reading instruction (5-min), then focusing on reading instruction (45-min), and then considering cognitive restructuring with a parent check-in (15-min). When a child develops skills in cognitive structuring (usually after session 7), gradual exposures replace the cognitive restructuring activities. The gradual exposures should focus on reading anxiety and integrate social anxiety modules into the programme if appropriate. This session structure (relaxation strategy − reading instruction − gradual exposures + parent check in) continues until the child becomes confident independently creating and completing gradual exposure activities, or the child reaches the end of the intervention period. It is important to note that while the current study administered 3-sessions per week, the anxiety intervention tailored for poor readers could be administered in 1-session per week, which is in line with other evidence-based cognitive behavioural interventions (Lyneham et al., 2003).

### Limitations and future research

The outcomes of this research must be considered within the context of strengths and limitations. One consideration is sample size. This study was designed to provide a close examination of PRAX intervention effects in individuals. It was the equivalent of a Phase 1 clinical trial. The positive outcomes encourage the equivalent of a Phase 2 trial that will include a larger group of participants to increase statistical power. Ideally, this would include three large groups of children with comorbid problems with reading and anxiety who are randomly allocated to one of three groups: one that receives intervention for reading and anxiety PRAX modules, one for reading modules only, and one for anxiety modules only. Comparing the outcomes of these groups will reveal if integrated PRAX intervention has a larger effect than reading intervention alone or anxiety intervention alone on reading and anxiety, respectively.

A second consideration is that the WEST (Howard, Best & Nickels, 2015) use a by-item analysis to evaluate the effect of an intervention on an outcome. This approach presumes that all items in an outcome measure are administered at each test point. This was not the case for three standardised outcome measures that used discontinue rules (nonword and word reading fluency, reading comprehension). After consultation with a WEST author, we made the decision to assume that children would have failed all items beyond the discontinue point. This risked misrepresenting PRAX intervention effects. The outcomes of this risk appear minimal since (1) these standardised tests are carefully designed so that very few items presented after a discontinue point should be correct, and (2) three (of the four) relevant outcomes were not specifically trained and did not show statistically significant treatment gains. The fourth outcome was specifically trained and did show significant treatment gains.

A third consideration is that the analyses in this study involved multiple comparisons, which increases the risk of Type I errors. The fact that the PRAX intervention effects on reading and spelling were limited to skills that were specifically trained (i.e. were not randomly distributed throughout outcome measures), paired with the fact that the PRAX intervention had a similar effect on anxiety as previous studies (Hudson et al., 2009), argues against undue influence of Type 1 errors in this study. Nevertheless, future studies could reduce the risk of Type 1 errors by restricting outcome measures of PRAX interventions to those that assess skills that are specifically targeted by training modules.

A final limitation of this study is that it was not designed to shed light on the theoretical mechanisms underpinning the association between poor reading and anxiety. Specifically, it did not test if poor reading causes later anxiety, or if anxiety interferes with the ability to learn to read. A recent series of analyses of four large longitudinal databases suggests that reading is more likely to have a causal effect on anxiety than vice versa (McArthur, Badcock, Castles, & Robidoux, under final revision with Reading Research Quarterly). It would be very useful if future intervention studies could tackle this issue directly.

### Summary

This study aimed to evaluate the efficacy of the first PRAX intervention in seven children with poor reading and anxiety. We found that the PRAX intervention significantly improved several skills related to reading accuracy, and significantly reduced anxiety disorders and symptoms. This justifies further testing of PRAX intervention for children with concurrent poor reading and anxiety in a larger randomised controlled trial.

## Supplemental Information

10.7717/peerj.10987/supp-1 Supplemental Information 1 Children’s standardised test scores for reading/spelling accuracy, reading fluency, reading comprehension, spoken language, and nonverbal intelligence at T1.

10.7717/peerj.10987/supp-2 Supplemental Information 2 Individual case studies, double-baseline.

10.7717/peerj.10987/supp-3 Supplemental Information 3 PRAX raw data.

10.7717/peerj.10987/supp-4 Supplemental Information 4 BAC data.

10.7717/peerj.10987/supp-5 Supplemental Information 5 The modifications made to the Cool Kids programme to create Cool Reading.

## Additional Information and Declarations

### Data Availability

The following information was supplied regarding data availability: Raw data are available in Supplemental Files.
