# Supplementary material for: The effect of an integrated reading and anxiety intervention for poor readers with anxiety
Source: PeerJ. 2021 Feb 24;9:e10987. doi: 10.7717/peerj.10987 (PMC7912612; doi:10.7717/peerj.10987)
Supplement: Supplemental Information 1 [file peerj-09-10987-s001.docx]

**Appendix B**

| Table B1  *Children’s standardised test scores for reading/spelling accuracy, reading fluency, reading comprehension, spoken language, and nonverbal intelligence at T1* | | | | | | | |
| --- | --- | --- | --- | --- | --- | --- | --- |
|  | CL | YR | ZK | CG | FM | JA | RF |
| Age (years:months) | 10:3 | 9:0 | 8:0 | 8:11 | 9:2 | 8:5 | 9:5 |
| Grade | 4 | 3 | 2 | 3 | 3 | 2 | 3 |
| **Reading accuracy (z scores)** |  |  |  |  |  |  |  |
| Regular words | -2.17 | -1.89 | -1.48 | -2.62 | -1.89 | -1.50 | -1.89 |
| Nonwords | -2.31 | -1.93 | -1.06 | -2.37 | -1.25 | -2.29 | -1.20 |
| Irregular words | -2.12 | -2.16 | -2.51 | -1.87 | -2.00 | -1.63 | -2.08 |
| **Reading fluency (standard scores)** |  |  |  |  |  |  |  |
| Nonwords | 59 | 76 | 86 | 70 | 76 | 63 | 74 |
| Sight words | 67 | 59 | 66 | 69 | 65 | 82 | 71 |
| **Spelling accuracy** |  |  |  |  |  |  |  |
| Nonwords **(scaled scores)** | 3 | 5 | 5 | 3 | 6 | 4 | 4 |
| Irregular words **(z-scores)** | -2.07 | -2.10 | -1.50 | -1.98 | -1.18 | -2.23 | -0.88 |
| **Reading comprehension**  **(stanine scores)** | 2 | 1 | 1 | 3 | 1 | 3 | 2 |
| **Expressive vocabulary**  **(scaled scores)** | 9 | 6 | 8 | 8 | 6 | 4 | 12 |
| **Nonverbal intelligence**  **(standard score)** | 97 | 103 | 105 | 113 | 95 | 100 | 88 |
| *Note.* Shaded cells indicate scores more than 1 SD below the mean of the normative data | | | | | | | |
