# Supplementary material for: The effect of an integrated reading and anxiety intervention for poor readers with anxiety
Source: PeerJ. 2021 Feb 24;9:e10987. doi: 10.7717/peerj.10987 (PMC7912612; doi:10.7717/peerj.10987)
Supplement: Supplemental Information 2 [file peerj-09-10987-s002.docx]

**Appendix C**

**Individual Case Studies, double-baselines**

Serje Robidoux

09/05/2019

**The problem**

For each child, we have measurements on a particular test at three timepoints. This is a clinical intervention, so the there are two baseline measures at Time 0 and Time 8 wks (T1 and T2, respectively), then a third measurement after a further 8 weeks of intervention (so T3 is 16 wks). The test used is a normed test with known^^[[1]](#footnote-1)^^ standard deviation and test-retest reliabilities.

The logic of this design is that children’s scores will change with time, simply due to cognitive maturation. By measuring the amount of change from T1 to T2, we can get an index of how much this happens. Then that change can be removed from the change between T3 and T2, and the leftover bit can be attributed to the intervention.

**The questions**

Howard et al. (2014) describe two ways of thinking about these test scores. In both cases, the question we are asking is essentially the same: is the score at T3 larger than we expect based on the scores at T2 and T1? The difference is in what counts as “larger”, and that depends on what we think the measures at T1 and T2 represent.

The simpler scenario (COL, or COmpare Level according to Howard et al.) is relevant when there is no particular reason to think that T1 and T2 differ in any way, so that T1 and T2 are really just two measurements of the exact same thing. Any change from T1 to T2 is just due to the (un)reliability of the testing, and not to any maturation, practice, or other systematic influences. The advantage here, is that the average of two measurements will be more precise than any single measurement, so that provides a bit more certainty about the baseline test score.

In this case “larger” means larger than the T1 and T2 scores combined: “Is T3 larger than the average of T1 and T2?”

The second way of thinking about these scores is to assume that in addition to test-retest “noise”, the change from T1 to T2 is also a systematic one that is related to practice effects, general maturation, or something else. In this case, we assume that the change from T1 to T2 would also occur in the time between T2 and T3 even without intervention.

In this second sense, “larger” means not just larger than T2, but enough larger that it can’t just be attributed to maturation or time. “Is the change from T2 to T3 larger than the change from T1 to T2?”

**Why not just use Howard’s WEST-COL and WEST-ROC?**

Howard et al.’s methods make use of item-level data, which is useful when you’re conducting a reading test or other kind of “accuracy” or “speed” measure. e.g., when each item can be compared with each other in reasonable ways.

If your test doesn’t work that way, say in a social anxiety questionnaire where the intervals between items may not have comparable psychological meaning, the item-level methods are no longer appropriate. We can, however, take the overall scores for the scale and use that to derive some useful values. Note that unless the test-retest reliabilities of your measure are very very high, this results in a considerably less powerful approach than the item-level approach that Howard et al. have developed.

**The Math**

In both of the scenarios described above, we are essentially taking three random variables (the measurements at T1, T2, and T3) and combining them into a new random variable.

COL: $X_{COL}=T3-\frac{T1+T2}{2}$

ROC: $X_{ROC}=(T3-T2)-(T2-T1)$ which can be rewritten in the simpler form $X_{ROC}=T3-2\cdot T2+T1$

What this means is that if we can work out the mean and standard deviations of $X_{COL}$ and $X_{ROC}$ under the null hypothesis that there are no effects of treatment, then we can use the simple normal distribution rule that forms the basis of most null hypothesis testing.

$$X_{crit}=\overline{X}\pm z_{\alpha}\sigma_{X}$$

where $\overline{X}$ is the expected mean of $X$, $z_{\alpha}$ is the standard normal value associated with alpha-level $\alpha$, and $\sigma_{X}$ is the standard deviation of the $X$ scores.

The trick is calculating $\overline{X}$ and $\sigma_{X}$ under the null hypothesis.

**Distribution of the COL score**

First let’s consider the easier situation, where there is nothing about the test that would imply a change with time. For example, if you were indexing social anxiety scores 8 wks apart, practice effects aren’t relevant since there are no right or wrong answers, and maturation is unlikely to produce systematic changes on this short timescale.

What we want to know is whether the scores at T3 are larger than we would expect given the scores at T2 and T1.

One assumption I’m making for the rest of this treatment is that T1, T2, and T3 are all distributed as $N(\mu,\sigma^{2})$, where $\sigma^{2}$ is constant for all three tests. Under the null hypothesis, $H_{0}$, we further assume that treatment makes no difference to test scores. Consequently, test scores will have identical $\mu$s. (Another assumption is that the test-retest reliability isn’t changing so that the correlation between T1 and T2, and T2 and T3, and T1 and T3 are all the same.)

What we need to do is work out what the distribution of the COL score is:

$$X_{COL}=T3-(T1+T2)/2$$

**Mean of** $\boldsymbol{X}_{\boldsymbol{COL}}$

To get the expected mean, it turns out to be simply

$$\overline{X}_{COL}=E(T3)-E(T1+T2)/2$$

$$\overline{X}_{COL}=\mu-(\mu+\mu)/2=\mu-\mu=0$$

**Variance of Linear Combinations of Variables**

The standard deviation is just the square root of the variance, so this discussion will be about variances.

There are two well-known features of variances that we can take advantage of here.

First, the variance of a linear combination of variables is a function of the individual variances, and the covariance of the variables:

$$Var(aW+bV)=a^{2}Var(W)+b^{2}Var(V)+2abCov(W,V)$$

Where $Var(\cdot)$ and $Cov(\cdot,\cdot)$ are the variance and covariances respectively.

In a few paragraphs we’re also going to need the fact that covariances are bilinear, i.e.:

$$Cov(W+V,U)=Cov(W,U)+Cov(V,U)$$

or more generally,

$$Cov(aW+bV,cU)=acCov(W,U)+bcCov(V,U)$$

Together, these two rules mean that we can take any linear combination of variables, and the variance of the combination will be a linear combination of the variances of each variable, and the pairwise covariances. Trust me, that’s simpler than the alternative.

**Variance of** $\boldsymbol{X}_{\boldsymbol{COL}}$

In our case, we’re looking for $Var(X_{COL})$ where $X_{COL}=T3-\frac{T1+T2}{2}$. To make this more like the rules introduced above, it’s useful to think of $X_{COL}$ as the sum of $1\cdot T3$ and $(-\frac{1}{2})\cdot(T1+T2)$, in which case we can use the first rule above

$$Var(X_{COL})=Var(T3-\frac{T1+T2}{2})=1^{2}\cdot Var(T3)+(-\frac{1}{2})^{2}\cdot Var(T1+T2)+2\cdot(-\frac{1}{2})Cov(T3,T1+T2)$$

which simplifies to:

$$Var(X_{COL})=Var(T3)+\frac{1}{4}\cdot Var(T1+T2)-Cov(T3,T1+T2)$$

This still leaves us with some annoying instances of $T1+T2$, so we have to get rid of those using both of the rules above.

$$Var(X_{COL})=Var(T3)+\frac{1}{4}[Var(T1)+Var(T2)+2\cdot Cov(T1,T2)]-[Cov(T3,T1)+Cov(T3,T2)]$$

Next we take the assumption that T1, T2, and T3 all have the same variance $\sigma^{2}$, and that the test-retest reliabilities will be the same for each pair of testings, namely $\rho$. In this case it turns out that each variance will be $\sigma^{2}$ while each covariance will be $\rho\sigma^{2}$.

$$Var(X_{COL})=\sigma^{2}+\frac{1}{4}[\sigma^{2}+\sigma^{2}+2\cdot\rho\sigma^{2}]-[\rho\sigma^{2}+\rho\sigma^{2}]$$

If we group all of that into a simpler form, we end up with

$$Var(X_{COL})=1.5\sigma^{2}-1.5\rho\sigma^{2}=1.5\sigma^{2}(1-\rho)$$

**Distribution of** $\boldsymbol{X}_{\boldsymbol{COL}}$

Alright, great, so under the assumptions presented here, we now have that

$$X_{COL}\sim N(0,1.5\sigma^{2}(1-\rho))$$

Now that we have this, we can use the formula above for determining whether or not our observed $X_{COL}$ score is far enough away from the mean to safely reject the null hypothesis. Namely, we can say that it has to be at least this big:

$$X_{COL.crit}=0\pm1.96*\sqrt{1.5\sigma^{2}(1-r)}$$

Here I’ve assumed a two-tailed test with $\alpha=.05$. For a one-tailed test, replace $1.96$ with $1.65$. For other levels of $\alpha$, use the appropriate value from the normal distribution.

1. By “known” here, I just mean well-estimated with a large sample [↑](#footnote-ref-1)
