# Supplementary material for: The effect of an integrated reading and anxiety intervention for poor readers with anxiety
Source: PeerJ. 2021 Feb 24;9:e10987. doi: 10.7717/peerj.10987 (PMC7912612; doi:10.7717/peerj.10987)
Supplement: Supplemental Information 5 [file peerj-09-10987-s005.docx]

| **Appendix A**  Table A1  *The modifications made to the Cool Kids program to create Cool Reading* | | |
| --- | --- | --- |
| Module | Modifications |  |
| **Psychoeducation**  Goal: to teach children and parents about the nature, cause, and experience of anxiety | - Replaced written workbook explanations with cartoon videos that were introduced in session - Activity sheets were simplified by removing written content and using images - Activities were completed in session with the clinician, child, and parent |  |
| **Controlled breathing**  Goal: to teach children relaxation strategies to calm their physiological response to anxiety and to tolerate feelings of distress when anxious | - Introduced the activity using a cartoon video that children could practice in-session with the clinician - Breathing was completed immediately before commencing reading treatment each session - Written explanations of controlled breathing were replaced with images - Visual cues were used to remind children to practice breathing |  |
| **Cognitive restructuring**  Goal: To modify children’s unrealistic or maladaptive thoughts, expectations, and beliefs | - Replaced written workbook instructions using cartoon videos - Illustrative pictures were used to teach children the steps of cognitive restructuring - The illustrative pictures were used as prompts and reminders for children to practice cognitive restructuring - The terminology was simplified by changing “realistic and unrealistic” thoughts to “calm and worried” - The writing demands on the child were reduced by inviting parents to record their child’s responses or allowing children to draw pictures |  |
| **Gradual exposure**  Goal: to reduce anxiety by teaching children to repeatedly and gradually face their fears to encourage new learning. | - Cartoon videos were used to introduce the rationale for gradual exposure - Written content was removed from the child workbook - The writing demands for the child were reduced by asking the clinician or parent to write the child’s responses, or allowing the child to draw pictures - Gradual exposures were delayed until session 13 (week 5) to provide children the opportunity to improve reading skills and confidence reading to reduce any potential negative reading related exposures |  |
| **Child management strategies for parents**  Goal: to teach parent’s strategies to reduce the maintenance of their child’s anxiety | - No modifications were made to the treatment materials per se - The materials were introduced in a modified session format as described in column 3 - The frequency in which parents attended sessions was modified to include the last 10 to 15 minutes of each session - Session 1: Rewarding children for managing anxiety - Session 2: Anxiety parenting traps; Anxiety, misbehaviour, or typical child? - Session 6: Providing support without encouraging anxiety - Session 9: Change the conversation - Session 12: Build independence and confidence; Parent Action plan - Session 13: Fighting fear by facing fear - Session 15: Challenges to exposure - Session 18: Build independence and confidence; Parent Action Plan - Session 19: Parent action plan review - Session 34: Coping skills |  |
| **Social anxiety treatment components**  Goal: to teach children specific skills to reduce fears related to social anxiety | - We included the social anxiety treatment components from Cool Kids social into the program - These components were administered to children with social anxiety - No modifications were made to the treatment materials per se |  |
| **Social skills and confidence**  Goal: to teach children basic social skills through role play activities | - No modifications were made to the treatment materials |  |
| **Structured problem solving**  Goal: to teach children to identify problems, brainstorm solutions, and select and execute a solution | - No modifications were made to the treatment materials |  |
| **Dealing with bullying**  Goal: to equip children with strategies to help them cope with bullying | - No modifications were made to the treatment materials |  |
| **Progressive muscle relaxation**  Goal: to teach children another relaxation strategy to reduce physiological arousal | - No modifications were made to the treatment materials |  |
